# Antibody-free digital influenza virus counting based on neuraminidase activity

**DOI:** 10.1038/s41598-018-37994-6

**Published:** 2019-01-31

**Authors:** Kazuhito V. Tabata, Yoshihiro Minagawa, Yuko Kawaguchi, Mana Ono, Yoshiki Moriizumi, Seiya Yamayoshi, Yoichiro Fujioka, Yusuke Ohba, Yoshihiro Kawaoka, Hiroyuki Noji

**Affiliations:** 1 Department of Applied Chemistry, The University of Tokyo, 7-3-1 Hongo, Bunkyo-ku, Japan; 2 ImPACT Program, Cabinet Office, Government of Japan, Chiyoda-ku, Tokyo, 100-8914 Japan; 3 Division of Virology, Department of Microbiology and Immunology, Institute of Medical Science, University of Tokyo, Minato-ku, Tokyo, 108-8639 Japan; 4 Department of Pathobiological Sciences, School of Veterinary Medicine, University of Wisconsin-Madison, Madison, Wisconsin 53711 USA; 5 Department of Cell Physiology, Faculty of Medicine and Graduate School of Medicine, Hokkaido University, N15 W7, Kita-ku, Sapporo, Japan

## Abstract

There is large demand for a quantitative method for rapid and ultra-sensitive detection of the influenza virus. Here, we established a digital influenza virus counting (DIViC) method that can detect a single virion without antibody. In the assay, a virion is stochastically entrapped inside a femtoliter reactor array device for the fluorogenic assay of neuraminidase, and incubated for minutes. By analyzing 600,000 reactors, the practical limit of detection reached the order of 10^3^ (PFU)/mL, only 10-times less sensitive than RT-PCR and more than 1000-times sensitive than commercial rapid test kits (RIDTs). Interestingly, neuraminidase activity differed among virions. The coefficient of variance was 30–40%, evidently broader than that of alkaline phosphatase measured as a model enzyme for comparison, suggesting the heterogeneity in size and integrity among influenza virus particles. Sensitivity to oseltamivir also differed between virions. We also tested DIViC using clinical gargle samples that imposes less burden for sampling while with less virus titre. The comparison with RIDTs showed that DIViC was largely superior to RIDTs in the sensitivity with the clinical samples although a few false-positive signals were observed in some clinical samples that remains as a technical challenge.

## Introduction

Influenza is an annual global occurrence. The number of estimated deaths that are directly and indirectly attributed to influenza is 250,000 to 500,000. In addition, global pandemics that occur once every few dozen years have caused millions of deaths^1^. Influenza is caused by the influenza virus. The four viral types (A, B, C and D) infect both humans and/or animals. The influenza virus belongs to the *Orthomyxoviridae* family and has a genome consisting of eight single-stranded RNAs. The envelope of influenza displays three transmembrane proteins — proton channel (M2), hemagglutinin (HA) and neuraminidase (NA)^2,3^.

Anti-influenza drugs that target structural proteins of influenza virus are being actively developed^4^. Representative anti-influenza drugs include inhibitors of NA, as well as RNA polymerase inhibitors. The administration of these anti-viral drugs in the early stages of infection is expected to substantially reduce the number of deaths^5,6^. Such early treatment requires a rapid and highly sensitive method for the detection of influenza virus in the early stages of infection. In addition to swiftness and sensitivity, quantitative capability is also always required for influenza virus analysis. A highly sensitive and quantitative method for virus measurement is mandatory to quantify the efficacy of novel influenza vaccines and anti-viral drugs^7^.

The classic method for the detection of influenza virus is the plaque assay^8^, which enumerates the number of plaques of dead cells or antigen-positive cells resulting from the virus infection. The PFU/mL value measured with this assay is the standard in viral quantification. However, because it takes several days to form a plaque, this assay is not suitable for a rapid diagnostic test.

Immunochromatography-based tests, such as the lateral flow test, is the standard clinical diagnosis test for the detection of influenza virus. The various versions are termed rapid influenza diagnostic tests (RIDTs)^9,10^. The method is easy and inexpensive, and it is the main test in the clinical diagnosis of influenza virus. However, immunochromatography is not sufficiently sensitive to detect influenza infection, especially in the early stage. The sensitivity of immunochromatography-based RIDTs, the probability to give positive signal for the samples that were identified as positives with RT-PCR, is only 70%^9,11^.

To address these technical challenges, diverse analytical methods for the detection of influenza virus have been developed^12,13^. They typically require specific binding of probes to influenza virus particles. Examples include DNA aptamers, fluorescent beads, and metal nanoparticles^14^. Sensing technology is also being actively investigated. Examples include interferometry combined with nanochannel device^15^, surface enhanced Raman scattering^16^, diamond electrode^17^, and field-effect transistor^18^. However, these methods still face challenges. Methods that require probe attachment are always hampered by the nonspecific binding of the probe, which increases the background signal. Methods that require advanced devices, materials, or imaging systems can be limited in their accessibility and usability, which hampers their value in diagnostic testing.

As a newly emerging analytical method with a detection sensitivity of single molecules or single entities, the digital bioassay technology is being rapidly developed^19,20^. In this method, micron-sized reactors with a volume of a few femtoliters (fL) are prepared in a large number, and enzyme molecules for detection are stochastically entrapped in each reactor with fluorogenic substrate to generate fluorescent reaction products. Due to the small volume of the reactor, the fluorescent molecules rapidly accumulate upon the catalytic turnover to a detectable level in a short time. Another advantage of the digital bioassay is that it can reveal the variance of activity among molecules^21^.

The practical use of highly sensitive digital bioassays is being actively studied. The most widespread use of the digital assay is digital PCR^22–24^. Since signals are exponentially amplified in PCR, digital measurement is readily achieved even in relatively large reactors. Various types of digital PCR and similar nucleic acid detection methods have been described^25^. Another digital bioassay for which practical application is expected is digital enzyme-linked immunosorbent assay (ELISA). This requires smaller reactors with the volume in the fL range as it is based on enzymatic reaction that linearly enhances signal upon turnover. The first digital ELISA was reported in a system consisting of optical fibre bundle^26^. Subsequently, a fL reactor array device (FRAD) that displays a massive number of micron-sized droplet reactors was utilized to realize more sensitive digital ELISA^27^. It has become a standard method^28,29^. Microreactor device equipped with mechanical valves was also developed for performing all the operations of digital ELISA on chip^30^. The digital concept is versatile and applied for the activity measurement of membrane transporter proteins^31,32^, and the detection of bacteriophage^33^.

In this paper, we established a highly-sensitive quantitative method for the rapid detection of the influenza virus. Previously, digital ELISA assay for influenza virus was reported^34^. Although ultrasensitive detection of influenza virus was elegantly achieved, the protocol requires liquid handling processes involved in separation of bound and free detection antibody (B/F separation) as well as condensation of capture beads by centrifuge. To circumvent such cumbersome separation procedures, we employed the fluorogenic assay for NA activity of influenza virus. The present method allows extremely simple protocol that requires just mixing of analyte with fluorogenic substrate. This novel approach allows to study the intrinsic heterogeneity of native influenza particles in terms of activity of neuraminidase and sensitivity to a specific inhibitor.

## Results

### Fluorogenic NA assay

**Figure 1:**
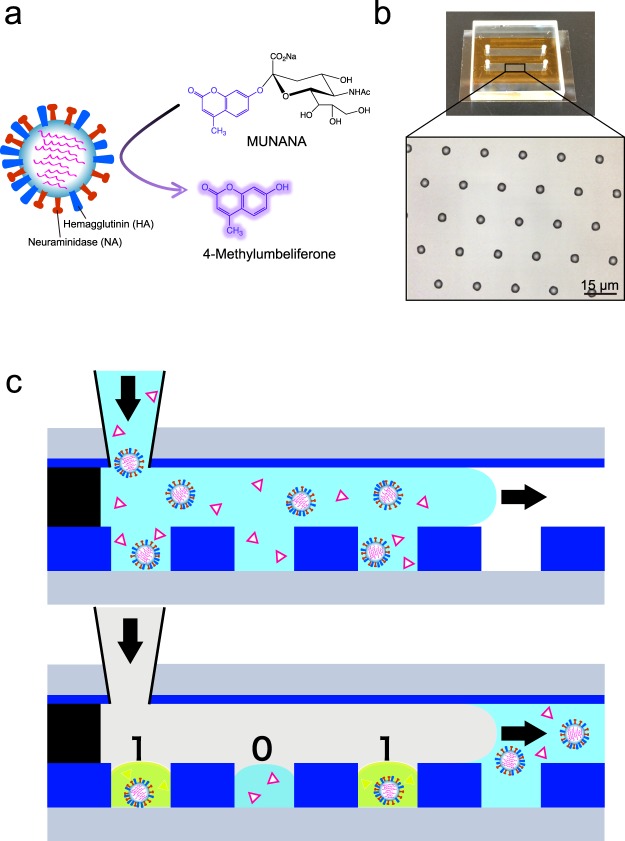
Diagram of the DIViC. (**a**) Schematic diagram of the fluorogenic assay for the neuraminidase (NA) activity of influenza virus. Influenza virions possess two surface glycoproteins, hemagglutinin (HA, shown in blue) and (NA, red). The fluorogenic substrate (2′-(4-methylumbelliferyl)-α-D-*N*-acetylneuraminic acid, MUNANA) is cleaved by NA to 4-methylumbelliferone (4-MU). Although MUNANA is non-fluorescent, 4-MU is fluorescent (excitation peak: 372 nm, emission peak: 445 nm). (**b**) Outlook and expanded image of a flow cell made of a glass plate (top), a spacer sheet (orange) and a fabricated device (bottom). The top cover glass has four access ports for sample injection and evacuation. The bottom coverslip has an array of CYTOP through-hole reactors (3.5 μm in diameter, 1.8 μm in thickness and 10 or 17 μm centre-to-centre distance) on its surface. A flow cell has two flow channels each having 600,000 reactors. (**c**) Schematic illustration of encapsulation of a single influenza virion in a reactor. Aqueous solutions containing influenza virus and MUNANA are injected into the microreactor arrays. Then, oil is injected to seal microdroplets in the reactors.

We performed a fluorogenic assay for neuraminidase (NA) of influenza, where 2′-(4-methylumbelliferyl)-α-D-*N*-acetylneuraminic acid (MUNANA) is hydrolysed to 4-methylumbelliferone (4-MU) and acetylneuraminic acid by NA activity (Fig. 1a). MUNANA is non-fluorescent, while 4-MU, which is a derivative of umbelliferone, is a fluorescent dye. The NA activity can be quantified from the rate of increase in fluorescence intensity. Before the digital assay, we performed a fluorogenic assay for influenza A/Puerto Rico/8/34 (H1N1) (hereafter referred to as PR8) in a conventional test tube using a fluorospectrometer. The activity was 2 × 10^4^ turnovers/s/PFU (Supplemental Fig. 1). This activity was 10-times higher than the activity of *Escherichia coli* alkaline phosphatase (*Ec*ALP), 1 × 10^3^ turnovers/s/molecule that was measured with an umbelliferone-based fluorogenic subustrate^35^.

### Digital influenza virus counting (DIViC) with FRAD

**Figure 2:**
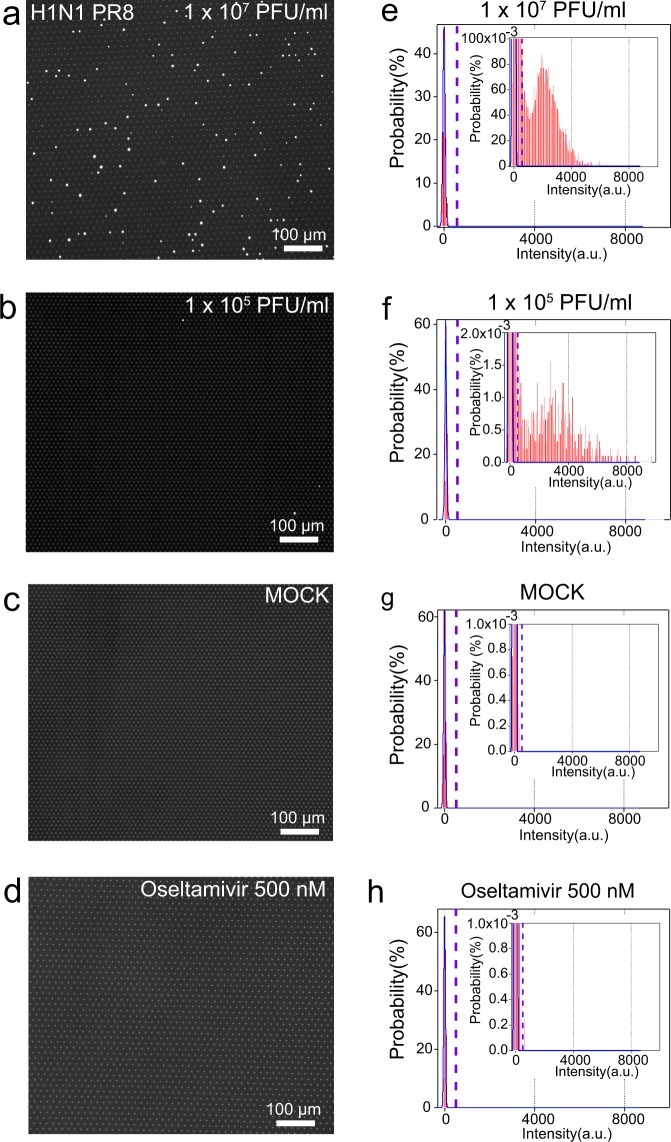
Fluorescence images of DIViC and distribution of fluorescence intensity. (**a**–**d**) Representative fluorescence images obtained in DIViC at different titres with 1 mM MUNANA. (**e**–**h**) Distribution of the fluorescence intensity of microdroplet reactors, with insets for the expanded graph. The blue lines indicate fits to the normal distribution to the first peak corresponding to empty reactors. The dotted lines show the threshold lines to discriminate positive reactors from empty ones. (**a**,**e**), and (**b**,**f**), Influenza virus (A/Puerto Rico(PR)/8/1934(H1N1)) 1.0 × 10^7^ and 1.0 × 10^5^ PFU/mL with 1 mM MUNANA. (**c**,**g**), Mock sample. Madin–Darby canine kidney (MDCK) cells without viruses. (**d**,**h**), Influenza virus (A/PR/8/1934(H1N1)) 5.0 × 10^6^ PFU/mL with 500 nM oseltamivir.

We performed a digital assay of influenza virus PR8 with a fL reactor array device (FRAD). The reactor was 3.5 μm in diameter and 1.8 μm in height. The resultant volume was 17 fL (17 × 10^−15^ L) (Fig. 1b). We measured about 600,000 reactors in each assay. We diluted a sample to a virus titre of 3.2 × 10^9^ PFU/mL and compartmentalized sample solution into FRAD (Fig. 1c). The sample was incubated for 10 min at room temperature and 5 min at 37 °C after sealing in the FRAD. The 10-min incubation was for bringing a device from the virus preparation room to the microscope room. Figure 2a shows an example of a fluorescent microscope image of reactors. Most reactors did not fluoresce, but some did present a fluorescence signal that was sufficiently clear. Dilution of samples (1:100) decreased the number of fluorescent reactors (Fig. 2b), while the fluorescence intensity of fluorescent reactors was constant regardless of the dilution rate (Fig. 2a,b). A mock sample without influenza virus did not fluoresce (Fig. 2c). When the NA inhibitor oseltamivir (Tamiflu^®^) was added, only very few fluorescent reactors showed weak fluorescence (Fig. 2d). We will discuss this point in detail below, in the section ‘heterogeneity of oseltamivir resistance’. These results confirmed that the bright fluorescent reactors are due to the neuraminidase activity of influenza virions.

We quantitatively measured the number of reactors that fluoresced by analysing the histograms of the fluorescence intensity of the reactors (Fig. 2e–h). First, we determined the distribution of back ground fluorescence signal (BGFS) from empty reactors that corresponds to the leftmost peaks in the histograms. BGFS is originated from the dark current of the camera and scattered light from the device. Because BGFS slightly differed among assays, BGFS was determined for each assays, by fitting the leftmost peak with a normal distribution. We defined the mean ± 15 × standard deviation (SD) of BGFS as the threshold for the discrimination of a positive fluorescence signal. When BGFS follows a normal distribution, the probability of exceeding this threshold is one in 10^50^ reactors, sufficiently low to avoid false-positive signals. When we measured three mock measurements, no positive reactors were identified among 1.8 million reactors. Next, we counted the numbers of positive reactors using 1.0 × 10^7^ and 1.0 × 10^5^ PFU/mL samples. The percentage of positive reactors (3.3% and 0.037%, respectively) was proportional to the values in PFU/mL (Fig. 2e,f). On the other hand, the mean fluorescence intensity was constant at approximately 2,500 arbitrary units (a.u.) irrespective of the virus titre. Thus, we confirmed three basic characteristics of digital bioassay—signals are quantized to zero and one, the proportion of positive reactors is proportional to the analyte concentration, and the signal value of positive reactors is constant and does not depend on analyte concentration. The confirmation of these characteristics in turn confirmed that a positive fluorescence signal represents the NA activity of single influenza virions.

With a calibration curve of 4-MU (Supplemental Fig. 2), we determined the mean enzymatic activity of a single virion to be 240 turnovers/s/particle from on the mean brightness of positive reactors. This value was about 100 times slower than the expected reaction rate per PFU from the bulk experiment, indicating that the actual number of virions in a sample was 100 times higher than the number based on PFU. We examined this point in detail in the following analysis.

### Comparison with PFU

**Figure 3:**
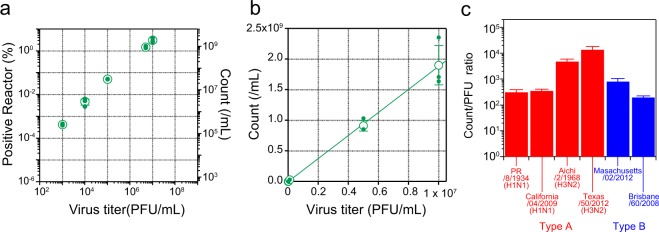
Counts of DIViC versus PFU. (**a**) Filled green circles indicate the fraction (%) of positive reactors that display a fluorescence signal exceeding the threshold value (mean ± 15 × SD). The right axis indicates the corresponding counts per mL. Open green circles are the averaged value from three replicates, and the error bars represent the standard deviation. (**b**) The linear-linear scale graph of (**a**). Green solid line indicates the fit with a linear function, giving the count-to-PFU ratio (CTPR). (**c**) CTPR of four subtypes of influenza A viruses and two lineages of influenza B viruses.

We diluted the PR8 samples ranging from 1.0 × 10^3^ to 1.0 × 10^7^ PFU/mL and plotted the positive reactor counts/mL against the virus titre. A fine linearity was observed over 4 orders of magnitude (Fig. 3a). This ensured that the DIViC method has a high quantitative performance that is similar to other digital bioassays.

The number of virus particles determined by DIViC was 100-times more than the number based on PFU at all concentrations. We plotted the data in a linear scale and obtained the ratio of positive reactor count to virus titre (count-to-PFU ratio, CTPR) from the slope as 189 counts/PFU (Fig. 3b). We also made the same measurements using different types and subtypes (Supplemental Fig. 3). Other subtypes of influenza A and two lineages of influenza B displayed CTPRs of several hundred, while the CTPR of type A H3N2 was extremely large, at 10,000 (Fig. 3c). The CTPR of H3N2 changed substantially depending on the preparation procedures (see Supplemental Fig. 4), perhaps reflecting low stability or higher sensitivity to preparation protocol. Other than the exceptional value of H3N2, CTPR was within the range of several hundreds, in consistent with other studies although a smaller value for particle-to-PFU ratio was also reported in some literatures (Supplemental Table 1)^36–41^.

As discussed above, no false-positive reactors were observed using mock sample, suggesting the practical detection limit is not determined by false-positive signal from BGSF. As shown in Fig. 3a, for samples of 1.0 × 10^3^ PFU/mL, on average, three of 600,000 reactors were positive (N = 3). Thus, a practical detection limit should be in the order of 10^3^ PFU/mL (10^5^ counts/mL). For comparison, we tested, with the same sample of PR, three commercially available RIDTs: Prorast Flu One (LSI Medience Corporation, Japan), QuickNavi Flu (Denka Seiken, Japan), and BD Flu Examen (Becton Dickinson, USA). In all tests, the lowest virus titre that gave recognizable signal in RIDTs were order of 10^7^ (PFU/mL) while not signal was recognized with the sample of 10^6^ (PFU/mL), suggesting that the detection limit of RIDTs is between 10^7^ and 10^6^ (PFU/mL). This is in consistent with the previously published range of detection limits of 10^4^ to 10^7^ PFU/mL although different virus types and subtypes were used in the paper^10^ where we assume that PFU/mL corresponds to 0.69 × TCID_50_/mL^42,43^. We also performed RT-PCR for the detection of PR8 with a protocol recommended by the World Health Organization and examined the detection sensitivity using the same sample of PR8. In this case, the detection limit was 4.2 × 10^2^ PFU/mL (8.0 × 10^4^ counts/mL) (Supplemental Fig. 5). As such, DIViC has the detection sensitivity that is only 10-times less sensitive than the normal RT-PCR method and which is 1000 to 10,000 times more sensitive than the RIDTs for the detection of PR8. It should be noted that the sensitivity of DIViC for other subtypes would differ.

### Heterogeneity of NA activity

**Figure 4:**
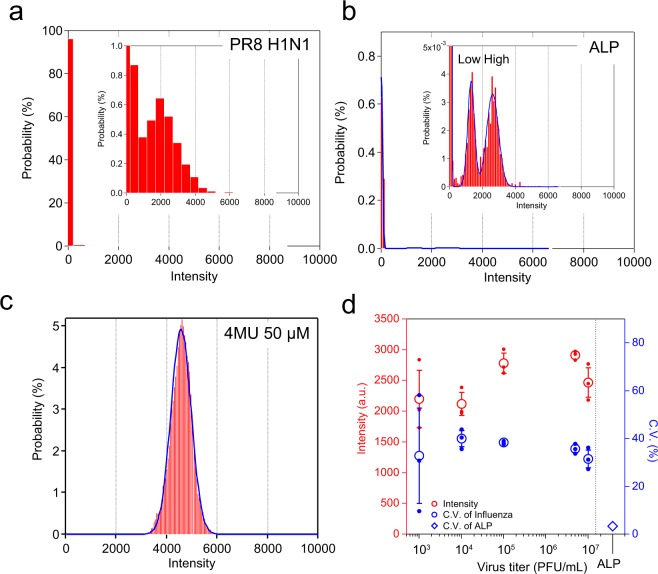
Variance in catalytic rate of influenza virus particles. (**a–c**) Representative distributions of fluorescence intensity of the microdroplet reactors. (**a**) Influenza A virus (A/Puerto Rico(PR)/8/1934(H1N1)) at 10^7^ PFU/mL. (**b**) Bovine alkaline phosphatase (ALP). Blue line indicates the result by fitting with gaussian mixture model. (**c**) 4-MU (50 μM) for the estimation of measurement noise level. (**d**) Red open circles indicate the average intensity of the positive reactors at various virus titre in the range from 10^3^ to 10^7^ PFU/mL. Blue open circles indicate the average C.V. of neuraminidase (NA) activity after corrected with measurement noise determined from **c**. The blue diamond shows the C.V. of activity of ALP after error correction.

Compared with the digital assay for enzyme assay, a characteristic of the NA activity of influenza virions is the broad distribution of enzymatic activity (Fig. 4a,b shows the activity distribution of a single molecule for bovine ALP measured for comparison. ALP has molecular species with high and low activities, which produce two distinct peaks. In the present study, we used the activity of the high active population as a reference. The fraction of ALP with higher activity showed an evidently sharp distribution with a mean activity at 4,001 (A.U.) and a standard deviation of 359. The resultant coefficient of variance (C.V.) was 9.0%. The ALP fraction with lower activity displayed a similar C.V. (7.8%). These values were very close to the systematic measurement error (7.1%) that was determined from the fluorescence intensity of 50 μM 4-MU in FRAD (Fig. 4c). When corrected for the measurement error, the actual C.V. of ALP was estimated to be only 3.1% (blue open diamond in Fig. 4d). On the other hand, the influenza NA activity was approximately 37% (Fig. 4d). Even when corrected with the system noise, it remained high, at approximately 36%. As such, compared to a conventional enzyme, influenza virions displayed much broader variance in NA activity. For both ALP and influenza, the turnover number (*N*_t_) of the enzyme reaction exceeded 10^5^. Thus, this variance could not be attributed to the Poisson noise of reaction turnover number among molecules that is represented as $1/\sqrt{{N}_{t}}$, meaning that influenza virus poses an intrinsic heterogeneity of the NA activity among virions.

### Heterogeneity of oseltamivir resistance

**Figure 5:**
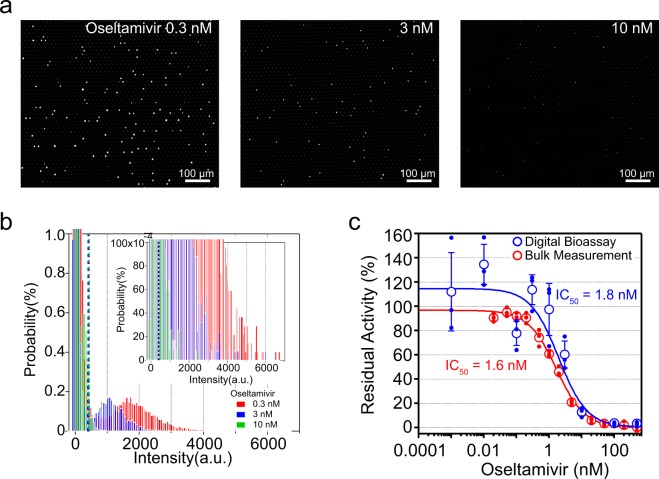
Inhibition assay with oseltamivir. (**a**) Representative fluorescence images of DIViC of influenza A virus (A/PR/8/1934(H1N1)) at 5.0 × 10^6^ PFU/mL with 0.3 nM, 3 nM or 10 nM oseltamivir. (**b**) Distribution of the fluorescence intensity with 0.3 nM (red), 3 nM (blue) or 10 nM (green) oseltamivir. Red, blue and green dotted lines indicate the threshold lines to define positive reactors at 0.3 nM, 3 nM and 10 nM oseltamivir, respectively. The top-right insets show expanded graph. **c**, Residual activity versus oseltamivir concentration. Red and blue filled circles indicate the residual activity measured in bulk experiments and DIViC, respectively. Open circles indicate average residual activity. Error bars represent the standard deviation. Data points were fitted according to competitive inhibition model to determine the half-maximal inhibitory concentrations (IC_50_) to be 1.6 nM for bulk measurements and 1.8 nM for DIViC.

To examine the inhibitory effect of oseltamivir, we conducted DIViC measurements of PR8 at various concentrations of oseltamivir. Figure 5a shows a representative fluorescence microscope image. As the oseltamivir concentration increased, the fluorescence intensity decreased. In addition, the number of reactors that could be confirmed as bright points also decreased. Figure 5b is a histogram of the fluorescence intensity. The mean fluorescence intensity decreased depending on the oseltamivir concentration. At an oseltamivir concentration of 10 nM, peaks could not be clearly separated from the BGFS distribution derived from empty reactors. At the oseltamivir concentration of 10 nM or higher, the distribution overlapped with the BGFS. In these cases, we selected reactors in the order of fluorescence intensity as much as the total number of viruses, and determined the mean intensity of the selected reactors (Fig. 5c). We obtained the IC_50_ of 1.8 nM by plotting the mean fluorescence intensity against the oseltamivir concentration. For comparison, we performed a fluorogenic assay with the same NA activity in bulk measurements to determine the IC_50_ of oseltamivir. The determined IC_50_ of approximately 1.6 nM was essentially consistent with IC_50_ values determined from DIViC and a previous report^44^.

**Figure 6:**
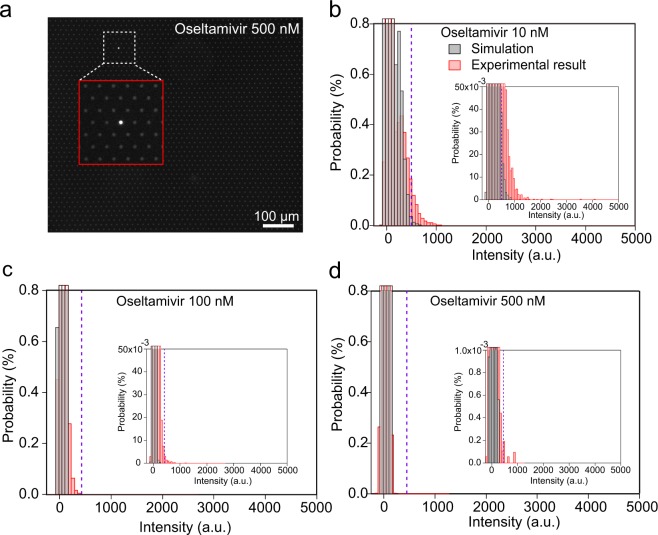
Simulation for oseltamivir inhibition. (**a**) Fluorescence images of DIViC of influenza virus (A/PR/8/1934(H1N1)) at 5 × 10^6^ PFU/mL with an excess amount of oseltamivir (500 nM). Enlargement of the image in the red box corresponding to the white dashed box. (**b**–**d**) The simulated distribution with the experimental data measured at 10 nM, 100 nM, and 500 nM. The black and red distribution indicate simulated and experimental one, respectively. The purple dotted lines show the threshold lines to discriminate positive reactors from empty ones.

Interestingly, even at concentrations significantly higher than the IC_50_, there were some fluorescent reactors. Figure 6a shows a fluorescence microscope image of the reactor observed at 500 nM oseltamivir, which was 300-times higher than the IC_50_. Under this condition, the activity should be supressed down to 0.3%, and any positive reactors should not be identified as positive reactors. Nevertheless, there were still several reactors found with signals higher than the threshold. In the three independent experiments at 500 nM oseltamivir, there were always several reactors identified as positive. In addition, although values were lower than the threshold, there were many reactors with fluorescence intensity very close to the threshold. In the mock measurement, there was no reactor with this brightness. Therefore, it is quite likely that these are also positive.

Here, we ask whether the heterogeneity of susceptibility to oseltamivir among virions should be considered or not. The alternative explanation is that apparent heterogeneity of inhibitor-susceptibility is not due to actual heterogeneous inhibitor-susceptibility but to different catalytic power among virions. In this a case, all of virions should be equally susceptible to oseltamivir with the common IC_50_ value, 1.8 nM. Then, the activity distribution in the presence of oseltamivir should be reproduced from the oseltamivir-free distribution (red one in Fig. 2e). To test this contention, we performed a simulation (Supplemental Fig. 6), by multiplying the oseltamivir-free distribution by the inhibition factors estimated from determined IC_50_: 13.9%, 1.6%, and 0.3% for 10, 100, and 500 nM oseltamivir, respectively. Figure 6b–d shows simulated distributions with experimental ones at 10, 100, and 500 nM oseltamivir. There are clear differences between simulated ones and experimental ones at three concentrations. Compared to simulated distributions, the experimentally obtained distributions are broader with brighter reactors. Thus, the observed distributions in the presence of oseltamivir are not explainable only by the heterogeneity of catalytic activity, implying that the heterogeneity of oseltamivir-susceptibility among virions exists.

### Evaluation with a clinical sample

**Figure 7:**
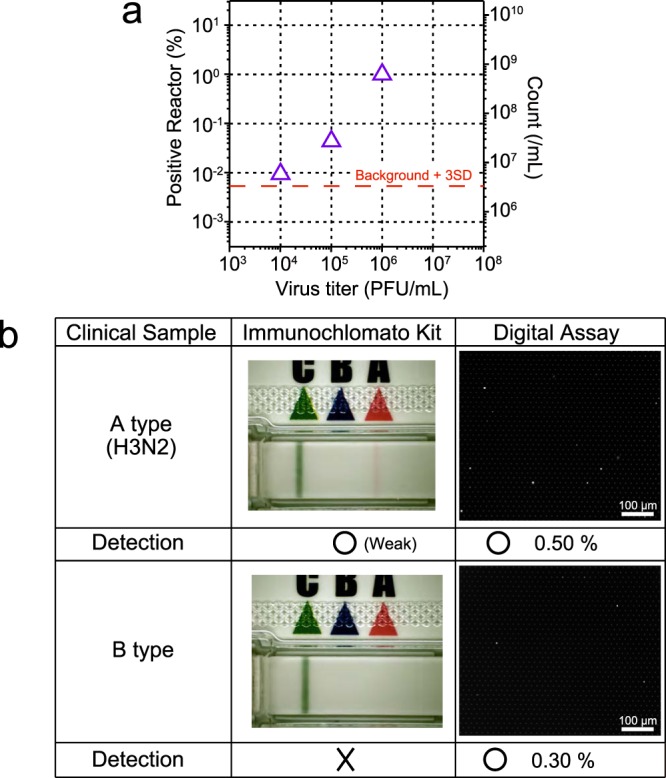
Clinical sample test with DIViC. (**a**) Spike experiments of DIViC with the clinical gargle sample from a healthy volunteer. The gargle sample was spiked with the indicated concentration of influenza virus (A/PR/8/1934(H1N1)). Purple triangles indicate the counts at various PFU concentrations. Red broken line represents background from contaminants of unknown origins in the gargle sample plus 3 times standard deviation that determined the limit of detection (LOD) at 3.4 × 10^6^ count/mL. (**b**) DIViC (right) and RIDTs (left) of gargle from patients.

Finally, to examine the potential of DIViC as a diagnostic assay, we evaluated the performance of the method using clinical samples obtained from patient whose nasal swab was identified as negative or positive by RT-PCR methods. Considering the superior sensitivity of DIViC over conventional RIDTs, we tested the feasibility of DIViC assay with a clinical sample from patients’ mouth fluid (gargle) because the gargle can be sampled with much less burden for patients while influenza virus is much less abundant influenza virus in patient gargle. Unlike pure solutions, the clinical sample has components derived from patients’ mouth fluid. Thus, measurement results may be influenced by the contaminants. We took measurements using a sample of a gargle solution from a healthy person and spiked it with PR8 sample (Fig. 7a). Similar to Fig. 3b, there were 592 times more fluorescent reactors compared to PFU. The mean and variance of the fluorescence intensity were almost the same as those without a gargle sample. On the other hand, measurements with mock gargle samples detected (1.9 ± 0.5) × 10^6^ counts/mL of unknown origin. The false-positive signal from gargle fluid samples was estimated to be 3.4 × 10^6^ count/ml. Thus, the sensitivity of DIViC for analysis of clinical sample is limited by the false-positive originated from contaminants in gargle. Even then, the sensitivity was still 1,000-times higher than the existing RIDTs.

Next, we performed DIViC of clinical gargle samples from patients who were identified as influenza-positive in RIDT tests of their nasal swab samples. For comparison, we also used a commercial RIDT (Fig. 7b). DIViC detected 2.9 × 10^8^ counts/mL and 1.7 × 10^8^ counts/mL with the gargle samples from Type A H3N2 or type B patient, respectively. On the other hand, RIDT detected only weak signal of Type A H3N2 while no signal was detected for type B. Thus, DIViC is superior in detection sensitivity to RIDT method even with clinical samples of less-burden for sampling. The signal with DViC was almost 100-times higher than the noise count for clinical gargle samples in DIViC. Thereby, detection would be still possible even when virus is 100-times less-abundant, suggesting that DIViC is applicable for diagnosis of early stage infections.

## Discussion

In this study, we combined a fluorogenic assay for neuraminidase (NA) and FRAD technology to establish the antibody-free digital influenza virus counting (DIViC) method that quantifies single influenza virions with ultra-high sensitivity. One of the main reasons for the high sensitivity is that the fluorescent signal of NA activity is significantly higher than the background fluorescence signal (BGFS) due to the high catalytic activity of NA as well as the small volume of reactors that condenses the signal spatially. The high fluorescence signal ensures high signal-to-noise (S/N) ratio of positive reactors to empty reactors that reached approximately 76. Due to such high S/N ratio, an extremely small number of fluorescent reactors can be accurately counted among a massive number of empty reactors. Even if only one reactor out of 600,000 is fluorescing, we can detect the presence of the virus. Mock samples showed no false-positive reactors among 1,800,000 reactors. Thus, the practical detection limit was approximately 10^5^ counts/mL. Considering that the count-to-PFU ratio was approximately 100, this was equivalent to 10^3^ PFU/mL. Even when considering the statistical variance, the practical limit of detection should be in the order of 10^3^ PFU/mL. This value is almost comparable to the detection sensitivity of RT-PCR (4.2 × 10^2^ PFU/mL) performed according to the World Health Organization recommended protocol. The DIViC method was 10^3–4^ times more sensitive than commercial RIDTs. In this study, we used the PR8 strain, which is often used as a reference. Similar results were also obtained with type A and type B influenza viruses. Thus, regardless of the type or subtype, DIViC detects influenza virus at very high sensitivity.

The sensitivity of DIViC is remarkable, even when compared with reported digital ELISA assay for influenza virus^34^ that achieved the ultra-high sensitive detection limit (10^3^ counts/mL for purified sample, and about 10^5^ counts/mL for spike assay). This is because the digital ELISA method has several enrichment procedures such as centrifuge and active trapping of microbeads into microwells. The present study does not employ any enrichment process in order to circumvent elaborated liquid handling processes.

The observed NA activity per single virion, 240 turnovers/s is reasonable when considering the total number of catalytic sites per virion and reported catalytic rate per NA monomer. Influenza virions present about 50 tetrameric structural proteins with NA activity as spikes^45,46^. The NA monomer reportedly reacts at approximately two turnovers/s^47^. Assuming that NA activity has simple additivity, the activity per virion is estimated to about 400 turnovers/s, which is close to the present value of 240 turnovers/s.

The fine linearity of signal against virus virion is another advantageous feature of DIViC. As shown in Fig. 3a, the number of positive reactors shows fine linearity against virus virion for over 4 orders of magnitude that is not achievable with the most of existing methods such as RT-PCR or RIDTs. Moreover, this feature allows to estimate the absolute number of virus virions from the number of positive reactors without a calibration curve because the current protocol of DIViC does not include concentration procedures. A possible concern is that a large particle-to-PFU ratio and its variability among types or subtypes of influenza. The comprehensive analysis of DIViC in comparison with other quantitative methods such as electron microscopic analysis and quantitative RT-PCR would address these concerns.

Another characteristic of DIViC is swiftness. The measurement is carried out by simply mixing the specimen with the fluorogenic substrate and sealing the mixture in the FRAD. Thus, in principle, it is faster than ELISA and other techniques that require separation procedures to wash out unbound probes or antibody conjugates. In this experiment, it took 10 min to carry the samples in the devices from a virus preparation room to the microscope room. The detection took 15 min in total including incubation at 37 °C. In preliminary experiments, we conducted assays in which the samples were incubated on the microscope stage at room temperature from the beginning. The positive reactors fluoresced after only 3 min, yielding an S/N ratio exceeding 2.0 (Supplemental Fig. 7). Thus, the DIViC method is very rapid, with a speed that is comparable with or higher than current RIDTs.

We measured gargle samples to test applicability of DIViC for diagnostics test. We spiked a gargle sample from a healthy volunteer with PR8 virions. The gargle sample did not interfere with the NA activity of influenza virions. The fluorescent reactors produced the same result as same as in the assay with the purified sample, and the fraction of positive reactors was proportional to virus titre. However, contaminating fluorescent reactors of unknown origin were present in the mock samples. Although false-positive signals were found in only 0.0041% of reactors, it limited the practical detection sensitivity to 10-times lower than in the purified system.

Next, we performed assays using gargle samples from a patient identified to harbour type A (H3N2) or type B influenza by RIDT testing of nasal swabs. The DIViC assay identified a large number of fluorescence reactors for both virus types. With type A (H3N2), the virus titre was sufficiently high for RIDT detection, although the signal was very weak and near the detection limit. On the other hand, the DIViC method detected a significantly stronger signal (0.5% of reactors). In the case of the type B sample, the virus titre was low and undetectable with RIDT, while a sufficient number of fluorescent reactors (0.3% of reactors) were observed in the DIViC assay. Thus, the use of actual specimens provided further confirmation of the superiority of the DIViC method to the commercial RIDT in terms of sensitivity. Additionally, DIViC enables virus detection with minimally invasive samples, such as gargle fluid or saliva.

For the diagnostic application of DIViC, suppression of the false-positive signal from contaminants in gargle samples is necessary. Considering that other NA enzymes from various origins could recognize the fluorogenic substrate, the false-positive signal might be caused by contaminating enzymes from oral bacteria or cells of the patients. Substitution of the hydroxyl group at the 4-position of sialic acid with a methyl group enhances the specificity to influenza NA compared to other NA enzymes^48^. Such chemical modifications of the fluorogenic substrate are expected to suppress the false-positive count.

A common characteristic among digital assays is the linearity of the signals over a wide range of analyte concentrations. Theoretically, the dynamic range of the digital assay is one-tenth or less of the total number of reactors. Therefore, with 10 ^*N*^ reactors, the dynamic range is the *N*-1 power, if there is no false positive. In the present DIViC method with 6 × 10^5^ reactors, the count was proportional in the range of 10^3^ to 10^7^ PFU/mL, giving a 4-digit dynamic range (Fig. 3a). Such a wide dynamic range is a characteristic feature of the DIViC method, which is not observed in other RIDT methods. When wider linearity is required, one can attain it simply by analysing more reactors.

The number of fluorescent reactors observed in this experiment was much higher than the expected from PFU at all concentration ranges. The count-to-PFU ratio (CTPR) of PR8 was 189. We also examined three other strains of type A and two strains of type B in addition to PR8. Except for the extraordinarily large value for the Texas/50/2012 strain, CTPR values were in the range of several hundreds for all other strains. Thus, it is evident that there is a major deviation between PFU and the actual number of virions, regardless of the strain or subtype of influenza virus. Many studies reported similar results to this study^36–41^. The most thorough and notable comparison determined the number of influenza virions in the same sample with various methods that included EM and RT-PCR, and compared these values against those of PFU^36–38,40,41^. The authors reported that the actual number of virions was dozens to several hundred times higher than that determined by PFU. In a previous experiment that used a laser dark-field microscope, we also observed that the number of scattered spots derived from influenza virus PR8 was 1,000-times higher than predicted from PFU^39^. The unusually high count-to-PFU ratio of Texas/50/2012 might be due to the difference in the preparation method and stability.

Another important characteristic of digital bioassays is that it allows us to examine the non-uniformity of an analyte directly. Presently, we observed two types of non-uniformity. One was the NA activity itself. As shown in Fig. 4a, the PR8 strain presented a C.V. of 36%. This value was much higher than 3% observed for ALP measured in comparison. The present activity was averaged over a prolonged time (15 min) compared to an enzyme reaction of approximately 1 ms. Thus, the difference was not attributable to the statistical variance of the number of reactions. Such a variance of the catalytic reaction rate exists in some enzymes and is attributed to a factor termed dynamic disorder that supposedly arises from the dynamic structural polymorphism^49,50^. In typical cases of dynamic disorder, frequent transition in the catalytic rate is observed on a time scale of seconds to minutes. On the other hand, we did not observe obvious transitions of the catalytic rate in long time trajectories exceeding 15 min. Thus, dynamic disorder does not adequately explain the heterogeneity of NA activity.

A feasible explanation is the variation in the copy number of catalytic sites of NA due to the variation in the influenza particle size. Previously we used transmission electron microscopy (TEM) to examine influenza PR8 that was freeze-dried using the platinum-replica method^39^. The examination revealed considerable variation in the size of virions (C.V. of 17%). Correspondingly, the C.V. of the surface area distribution should be 34%, which is very consistent with the variance observed in the present study. When the number of NA reaction centre is proportional to the surface area, the NA activity distribution observed in the present study can be well explained due to virion size distribution.

It is also possible that DIViC counts not only mature virions with perfect integrity but also unmatured virions that lack some components such as genomic RNA or structural proteins. This also explains the large count-to-PFU ratio partly. However, it is unlikely that DIViC counts very small fractured fragments of virus with one or two catalytic sites of neuraminidase. In such a case, we should observe a Poisson-like distribution. Actual distributions show a Gaussian-like distribution (Fig. 2a) suggesting some uniformity. The high catalytic turnover rate per particle (240/sec) compared with the reported turnover rate for monomer neuraminidase (2/sec) also supports this contention.

Another heterogeneity suggested in the present study is the resistance to the NA inhibitor oseltamivir. Even in the presence of 500 nM oseltamivir, which is approximately 300 times higher than the IC_50_, several fluorescent reactors were still observed. This is equivalent to 0.6% of the total virions. At such high inhibitor concentration, the NA activity is theoretically suppressed to 0.3% of the original activity. The highly inhibitor-tolerant virions that is only 0.6% of total cannot be explained solely by the variance of NA activity. It is not clear whether resistance to an inhibitor arises from genetic non-uniformity or from state or structural polymorphism of NA. The intrinsic heterogeneity of influenza virions were reported elsewhere. It was reported that tens to hundreds of thousands of virions should contain several mutants having a mutation of HA with differing antigenicity^51,52^. This is due to the high mutation rate of the virus polymerase^53^. Furthermore, a point mutation in the NA protein confers the oseltamivir resistance^54^. Considering these findings, it would be reasonable to assume the heterogeneity of the inhibitor resistance is at least due to genetic variations of NA.

Other reports have described functional diversities of the influenza virus. For example, the existence of non-infectious virions and noninfectious cell-killing virions has been proposed^55–57^. Their existence has been indicated by quantitative infection experiments. The source of the non-uniformity originates and its physiological role are unknown. In the future, by combining the DIViC method with other techniques for single virion analysis, the specific mechanism of the heterogeneity of influenza virus may be elucidated.

## Material and Methods

### Materials

Influenza A virus (A/Puerto Rico/8/1934(H1N1), A/California/04/2009 (H1N1) pdm09, and influenza B virus (B/Massachusetts/02/2012 and B/Brisbane/60/2008) were prepared as previously described^58^. Influenza A virus (A/Aichi/2/1968 (H3N2)) was prepared as previously described^59^. For the preparation of Texas/50/2012(H3N2)), stock sample was first used for plaque isolation, and then virus sample with single plaque origin was used for virus preparation (Supplemental Fig. 4). 2′-(4-Methylumbelliferyl)-α-D-*N*-acetylneuraminic acid (MUNANA) (Life Technologies, USA), Fluorinert-FC40 (3 M), CYTOP (Asahi-glass, Japan), alkaline phosphatase recombinant, highly active (ALP; Roche, Switzerland), Fomblin Y-LVAC 25/6 (Solvay, Belgium), 4-MU phosphate (4-MUP; Sigma-Aldrich, USA) were purchased from the respective suppliers. Three RIDT—Prorast Flu One (LSI Medience Corporation, Japan), QuickNavi Flu (Denka Seiken, Japan), and BD Flu Examen (Becton Dickinson, USA)—were purchased from the respective suppliers.

### FRAD

The fL reactor array device (FRAD) was prepared as follows. A hydrophobic carbon-fluorine polymer (CYTOP; Asahi-glass) was spin-coated on a clean coverslip (24 × 32 mm) at 2,500 rpm for 30 s and baked at 80 °C for 10 min followed by 180 °C for 30 min. The thickness of the CYTOP layer was approximately 1.8 μm. The CYTOP-coated coverslip was spin-coated with a positive photoresist (AZ-4203; AZ Electronic Materials, USA) at 1,000 rpm for 30 s and baked at 100 °C for 5 min. Subsequently, photolithography was carried out with a mask structure with 2-μm holes, which were separated by 17 or 10 μm. The resist-patterned coverslip was dipped in a developer (AZ300MIF; Merck, Germany) for 90 s in a sonic bath. The coverslip was dry-etched with O_2_ plasma in a reactive ion etching system (RIE-10NR; Samco, Japan) to remove the exposed CYTOP. The substrate was then cleaned and rinsed with acetone and isopropanol to remove the photoresist layer remaining on the substrate. The resulting CYTOP-on-coverslip device had an array of exposed SiO_2_ patterns with a diameter of 3.5 μm, which each held a water droplet in the digital influenza assay. The volume of one microreactor was 17 fL.

### DIViC procedure in the FRAD

The flow cell was assembled from the FRAD and a non-fabricated SiO_2_ glass coated with CYTOP separated by approximately 80-μm double-sided tape. Influenza solution was diluted with assay buffer A (1 M DEA-HCl, pH 9.0, 4 mM CaCl_2_). Next, the MUNANA fluorogenic substrate was added at 1 mM, and the reaction mixture was immediately introduced into the flow cell. Then, perfluorocarbon fluid (FC40) was introduced to flush out the excess reaction mixture and form water-oil droplets. Subsequently, FC40 was replaced by Fomblin to prevent the evaporation of the water droplets. After incubation for 10 min at room temperature, the device was incubated for 5 min at 37 °C with a stage heater on an inverted microscope (Olympus IX83, Olympus Co.). After that, fluorescence images were obtained with a CMOS camera via a ×20 objective lens. In total, 80,000–1,000,000 microdroplet reactors were observed in one assay. An X-cite fluorescence illuminator (Excelitas, USA) was used for excitation of 4-MU (λ_ex_ = 372 nm, λ_em_ = 445 nm) in the reactors. The fluorescence images were analysed with Image J software (NIH, USA).

### Inhibition assay of influenza virus using oseltamivir

Before introduction to the flow cell, oseltamivir was added to the reaction mixture, and the mixture was incubated for 10 min at room temperature in an Eppendorf tube. Then, the mixture was introduced in FRAD as same as inhibitor-free experiment. The activities were estimated in as follows. At the oseltamivir concentration of 10 nM or higher, the distribution of positive reactors largely overlapped with that of the BGFS. In these cases, we selected the number of reactors equivalent to the total number of viruses in the order of the highest fluorescence intensity, and determined the mean intensity of the selected reactors. The IC_50_ was determined from the fitting the mean fluorescence intensities.

The reactions for bulk measurement were performed in a 29-μL reaction volume containing 1 M DEA, pH 9.0, 4 mM CaCl_2_, 1 mM MUNANA, and either 5.0 × 10^7^ PFU/mL influenza virus or no virus, in addition to the indicated concentrations of oseltamivir on a 386-well plate (Greiner; Bio-One, Austria). The mixture was incubated for 10 min at room temperature. Then, the 4-MU fluorescence product was quantified using a FlexStation3 microplate reader (Molecular Devices, USA) with excitation and emission wavelengths of 360 and 448 nm, respectively, at 37 °C every 5 min. The activity of viral NA was determined from the slope ranging from 30–60 min after preparing the reaction mixture. The activity in the absence of oseltamivir or influenza virus was defined as 100% and 0%, respectively.

### Digital enzyme assay for ALP in the FRAD

The digital enzyme assay for ALP was carried out in a flow cell assembled with FRAD and top cover glass. ALP was diluted with assay buffer B (1 M DEA-HCl, pH 9.0, 2 mM MgCl_2_, Tween-20 0.1%) to 2 pM. The 4-MUP fluorogenic substrate was added to 1 mM and the reaction mixed was immediately introduced into the flow cell. Fluorescence images were obtained as same as DIViC.

### Diagnostic tests using RIDTs

The three RIDTs — Prorast Flu One (LSI Medience Corporation, Japan), QuickNavi Flu (Denka Seiken, Japan), and BD Flu Examen (Becton Dickinson, USA) — were used according to each manufacturers’ procedure. Influenza virus (A/Puerto Rico/8/1934(H1N1)) (PR8) was serially diluted 10-fold to be 5.0 × 10^6^, 5.0 × 10^7^, and 5.0 × 10^8^ PFU/mL. Then, a nasal swab attached to each RIDT was soaked in the serially diluted PR8. The swab was soaked in extraction buffer in an attached tube. In addition, the tube with the soaked swab was firmly pinched to squeeze out PR8 from the swab. Finally, a nozzle to dispense the sample was attached to the top of the tube and the extraction buffer including PR8 was dispensed onto the sample placement area on each test kit. The result was observed by the naked eye.

## Supplementary information

Supplemental Figures and Table
