# Supplementary material for: Antibody-free digital influenza virus counting based on neuraminidase activity
Source: Sci Rep. 2019 Jan 31;9:1067. doi: 10.1038/s41598-018-37994-6 (PMC6355933; doi:10.1038/s41598-018-37994-6)
Supplement: Supplementary file 1 — Supplemental Figures and Table [file 41598_2018_37994_MOESM1_ESM.pdf]

Title

Antibody-free digital influenza virus counting based on neuraminidase activity

Authors

*#Kazuhito V. Tabata[1,2], #Yoshihiro Minagawa[1], Yuko Kawaguchi[1], Mana Ono[1], Yoshiki Moriizumi[1], Seiya Yamayoshi[3], Yoichiro Fujioka[5], Yusuke Ohba[5], Yoshihiro Kawaoka[3,4], and *Hiroyuki Noji[1,2]

#These authors contributed equally.

Affiliations

[1]Department of Applied Chemistry, The University of Tokyo, 7-3-1 Hongo, Bunkyo-ku, Japan

[2]ImPACT Program, Cabinet Office, Government of Japan, Chiyoda-ku Tokyo 100-8914, Japan

[3]Division of Virology, Department of Microbiology and Immunology, Institute of Medical Science, University of Tokyo, Minato-ku, Tokyo 108-8639, Japan

[4]Department of Pathobiological Sciences, School of Veterinary Medicine, University of Wisconsin-Madison, Madison, Wisconsin 53711, USA

[5]Department of Cell Physiology, Faculty of Medicine and Graduate School of Medicine, Hokkaido University, N15 W7, Kita-ku, Sapporo, Japan

***Corresponding authors**

Hiroyuki Noji and Kazuhito V. Tabata

Department of Applied Chemistry, The Univesity of Tokyo,

7-3-1 Hongo, Bunkyo-ku, Japan

tel. +81-3-5841-7252

fax. +81-3-5841-1872

email : hnoji@appchem.t.u-tokyo.ac.jp

kazuhito@nojilab.t.u-tokyo.ac.jp

Supplemental Figures and Table

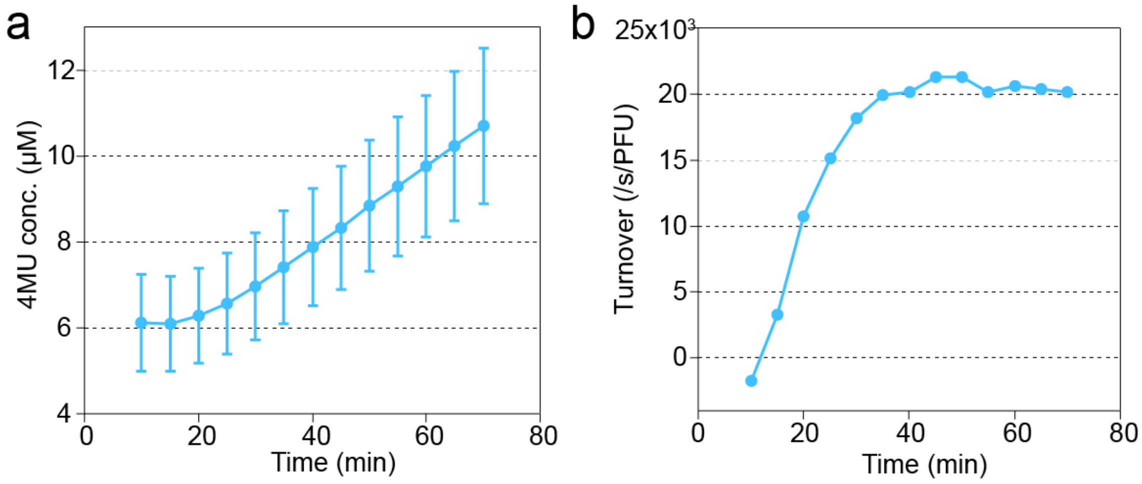

Supplemental Fig. 1. Fluorogenic assays of influenza virus in solution

a, Time courses of the fluorogenic assay of influenza virus. The reaction was measured in solution with the plate reader. The sample was influenza type A virus (A/PR/8/1934(H1N1)) at 1.0×10^7 PFU/mL mixed with 1 mM MUNANA. **b**, The determined turnover rate from the time course of (a).

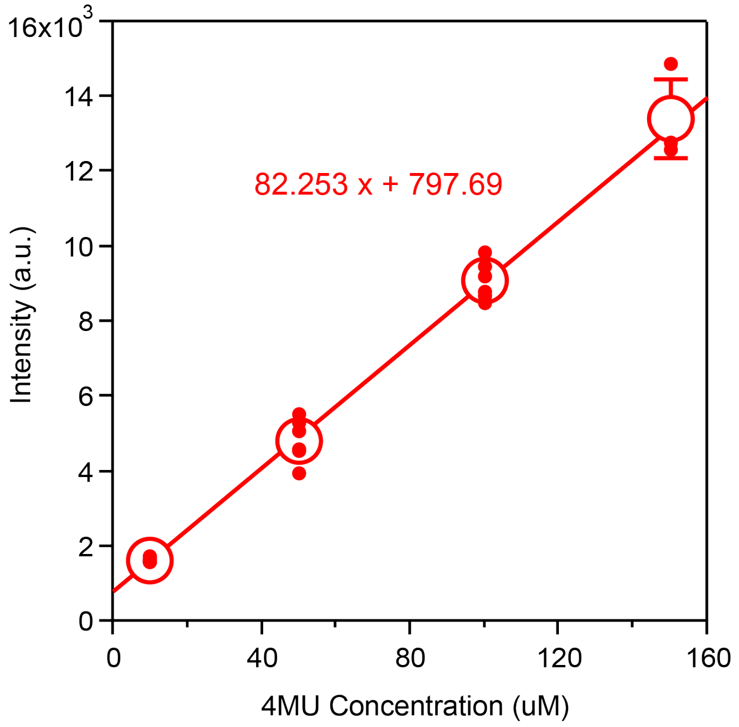

Supplemental Fig. 2. Calibration curve of 4-MU.
4-MU solution was partitioned into FRAD at the indicated concentrations. The fluorescent intensity of droplet reactors was determined under the measurement condition as same as in DIViC.

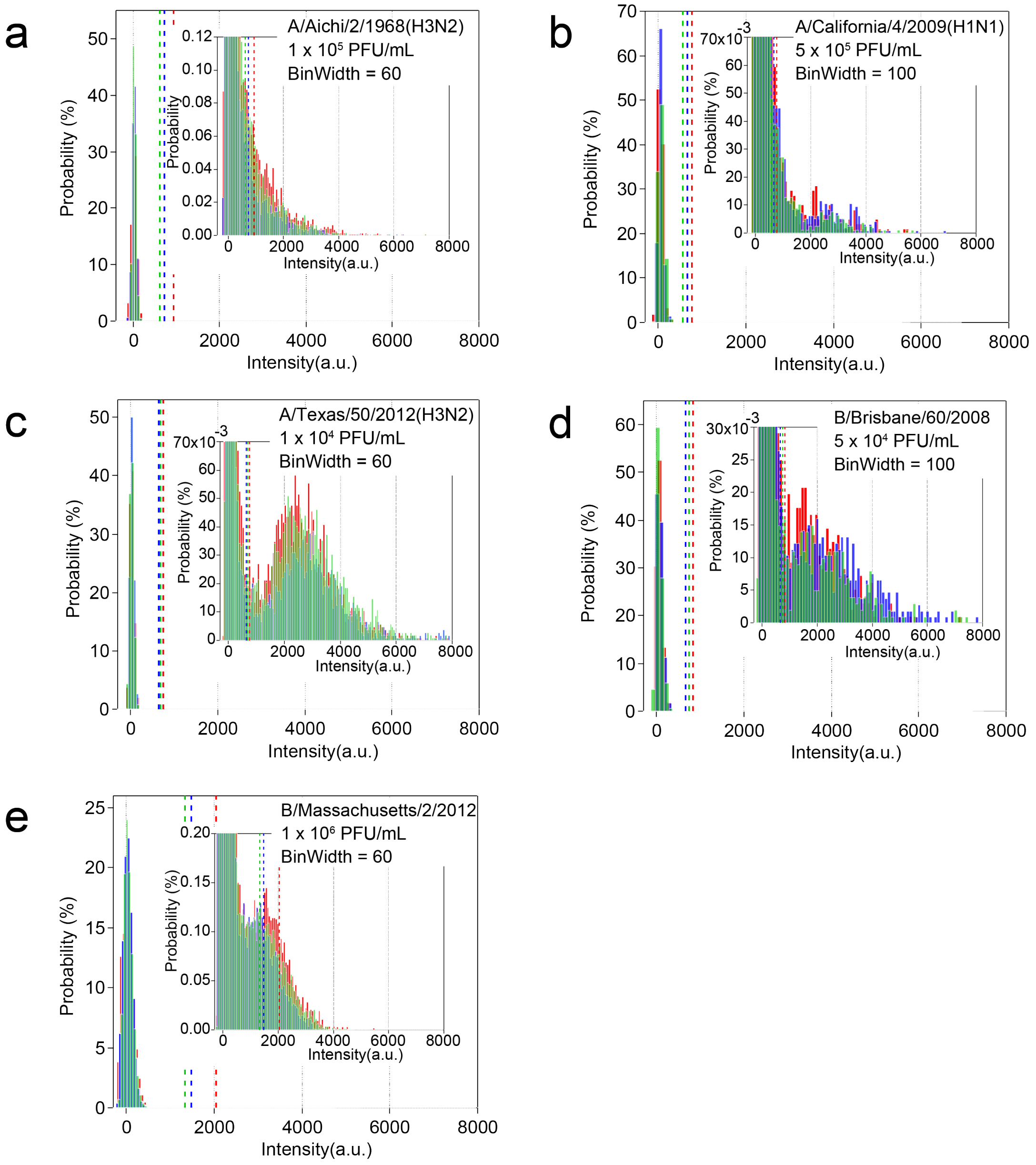

Supplemental Fig. 3. DIViC of various types and subtypes of influenza virus

Distribution of the fluorescence intensity of reactors from three replicates. The top-right insets show the expanded graph. **a,** A/Aichi/2/1968(H3N2), 1.0 × 10^6 PFU/mL. **b,** A/California/4/2009(H1N1), 5.0 × 10^5 PFU/mL. **c,** A/Texas/50/2012(H3N2), 1.0 × 10^4 PFU/mL. **d,** B/Brisbane/60/2008 (Victoria lineage), 1.0 × 10^6 PFU/mL. **e,** B/Massachusetts/2/2012 (Yamagata lineage), 5.0 × 10^4 PFU/mL.

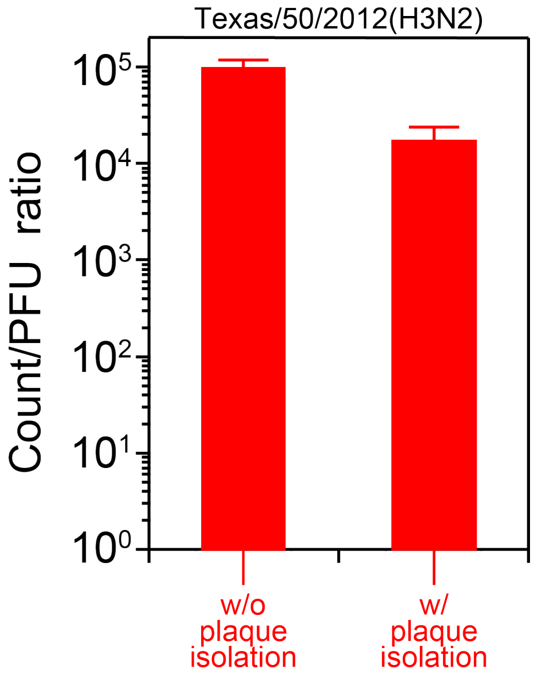

Supplemental Fig. 4. CTPR of H3N2 depending on the preparation procedures

Count-to-PFU ratio (CTPR) of Texas/50/2012 strain was determined for the preparation with or without plaque isolation. In 'w/o' preparation, a confluent MDCK cells on a dish plate were directly infected with a stock sample of Texas/50/2012, and the supernatant of the culture medium was recovered for analysis as same as for other strains. This preparation gave a large CTPR (left). In 'w/' preparation, the stock sample was firstly used for plaque assay with MDCK cells. A virus sample was recovered from a single plaque, and then used for the virus preparation. This preparation gave almost 10-time lower CTPR (right) and shown in the Fig. 3c.

Supplemental table 1. CTPR of PR8 reported in literatures

Literatures were surveyed that reported count-to-PFU ratio (CTPR) of A/PR/8/34 (PR8) strain for the comparison with the present study. The reported values for other subtypes were also provided.

Reference	Virus type	Count to PFU ratio	Method for particle counting
van Elden, L. J. R., et. al. Journal of Clinical Microbiology 39, 196-200, (2001). Ref. 36	**A/PR/ 8/34** (H1N1)	**750**	Electron microscopy (EM)
	B/Lee/ 40	**2357**	EM
Transfiguracion, J., et. al. Vaccine 33, 78-84, (2015). Ref. 37	A/Wilson Smith/1933 (H1N1)	**2**	EM
	B/Lee/1940	**> 50**	EM
	A/Aichi/2/68 H3N2	**> 50**	EM
	A/Hong Kong/8/1968 (H3N2)	**> 50**	EM
	**A/PR/8/34** (H1N1)	**3**	EM
Ward, C. L. et al. Journal of Clinical Virology 29, 179-188, (2004). Ref. 38	A/Shangdong/3/93 (H3N2)	**204**	Real time qPCR
	A/Taiwan/1/86 (H1N1)	**2381**	Real time qPCR
	B/Lisbon/3/96	**9091**	Real time qPCR
Enoki, S. et al. PLoS One 7, e49208, (2012). Ref. 39	**A/PR/8/34** (H1N1)	**< 2000**	TIRFM and SEM
K.H. Chan, et.al. J Clin Virol, 45, 3,2009 Ref. 40	A/HK/415742/09 (H1N1)	**909**	Real time qPCR
	A/California/4/09 (H1N1)	**591**	Real time qPCR
	A/HK/403946/09 (H1N1)	**591**	Real time qPCR
Noton et al. J Virol, (2009) Ref. 41	**A/PR/8/34** (H1N1)	**8**	EM
	**A/PR/8/34** (H1N1)	**37**	EM
	A/PR/8/34 ts and NP mutant	**500**	EM
	A/PR/8/34 ts and NP mutant	**1481**	EM

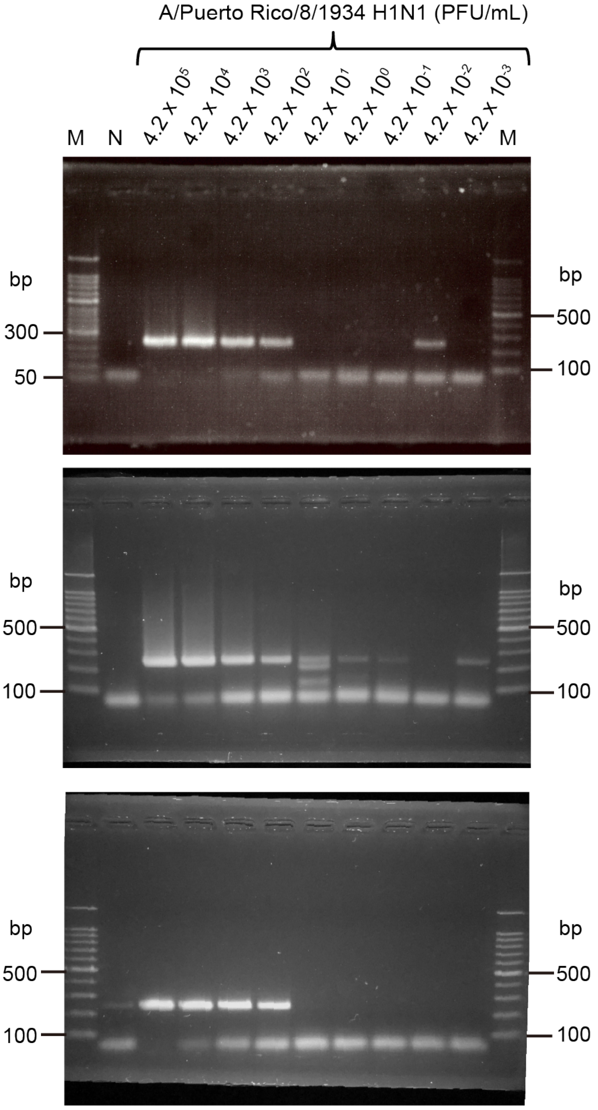

Supplemental Fig. 5. Limit of detection for conventional reverse transcription PCR

Three independent replicates of the virus detection with reverse transcription PCR (RT-PCR). Three different full-length agarose gels showed the results of RT-PCR amplicons for detection of matrix protein (MP) gene of influenza. The gel images were taken by WAT-120N$^+$ (1st experiment) or iPhone Camera (2nd, 3rd) and converted to gray-scale by Adobe® illustrator® CC. RT-PCR amplification was done using the conventional RT-PCR protocol based on *WHO information for molecular diagnosis of influenza virus*. In that protocol, forward-primer (M30F1/08 : 5'-ATGAGYCTTYTAACCGAGGTCGAAACG-3') and reverse-primer (M264R3/08 : 5'-TGGACAAANCGTCTACGCTGCAG-3') were used to detect MP gene of influenza type A virus (A/PR/8/1934(H1N1)(PR8). Expected PCR product size was 244 bp. Products were analysed by 2 % agarose gel electrophoresis and SYBR safe^{TM} gel staining. The PR8 strain was serially diluted 10-fold before extracting RNA. Lane 1, 50-bp (1st experiment) or 100-bp (2nd, 3rd) DNA ladder (TaKaRa, Japan). Lane 2, RNase free water. Lanes 3-11, serially diluted PR8 strain. Lane 12, 100-bp DNA ladder (TaKaRa). Although the second experiment detected virus at titre of 4.2×10^1 or less, the result was not reproducible in 1st and 3rd experiments and non-specific amplification was observed in the second experiment. Considering the reliability, the detection limit in the present condition was judged as 4.2×10^2.

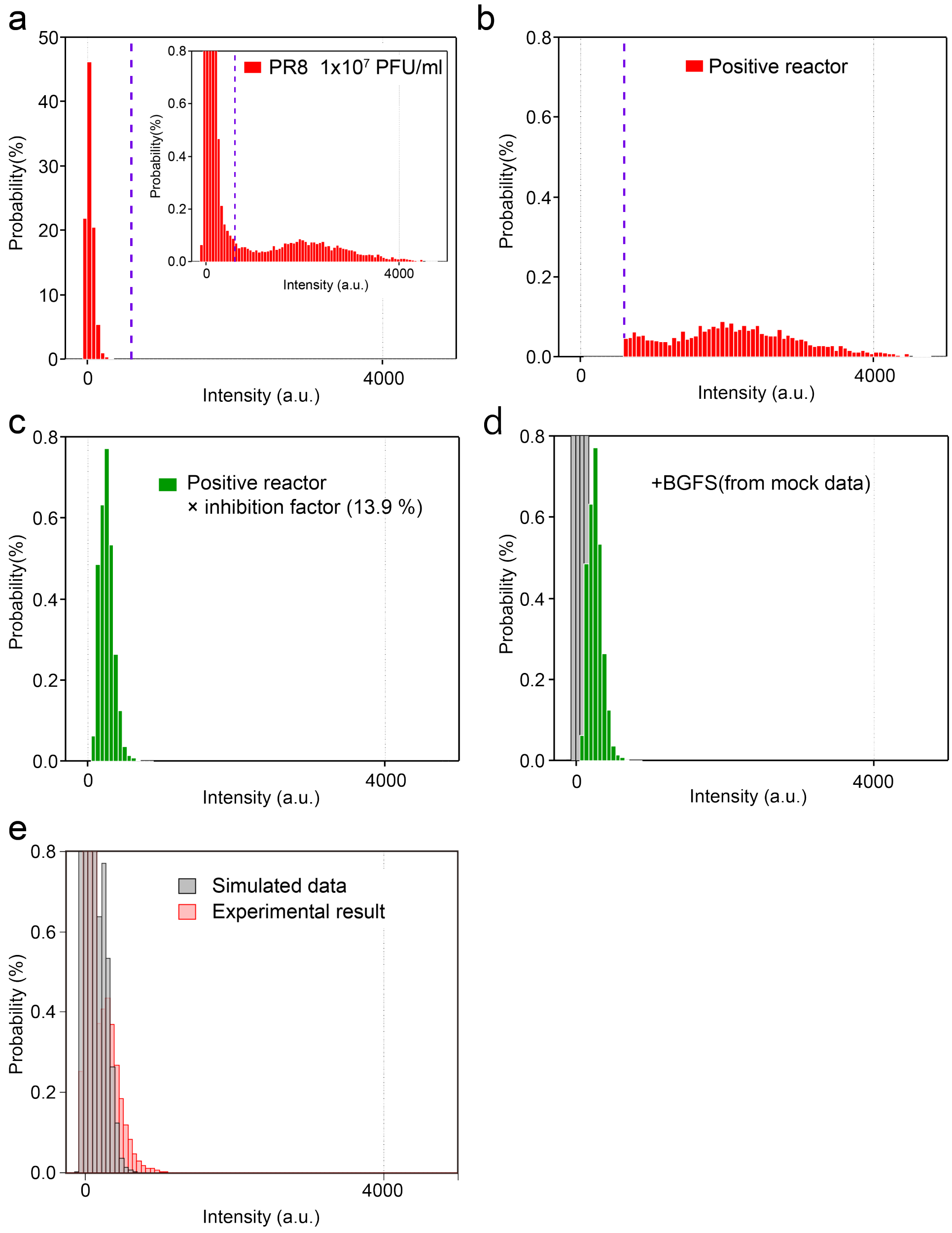

Supplemental Fig. 6. Simulation procedure for oseltamivir inhibition

a. The original distribution of PR8 at 5.0 × 10^6 PFU/mL in the absence of oseltamivir. The dotted vertical line is the threshold to define positive reactors. **b.** Distribution after removing background fluorescence data in **a**. **c.** calculated distribution at 10 nM oseltamivir that is obtained by multiplying the extracted distribution (**b**) with the inhibition factor (13.9%) estimated from IC_{50} of 1.6 nM. **d.** the simulated distribution obtained from the calculated distribution plus the background fluorescence measured in mock sample. **e.** the comparison of the simulated distribution with the experimental data measured at 10 nM oseltamivir that is shown as Fig. 6b.

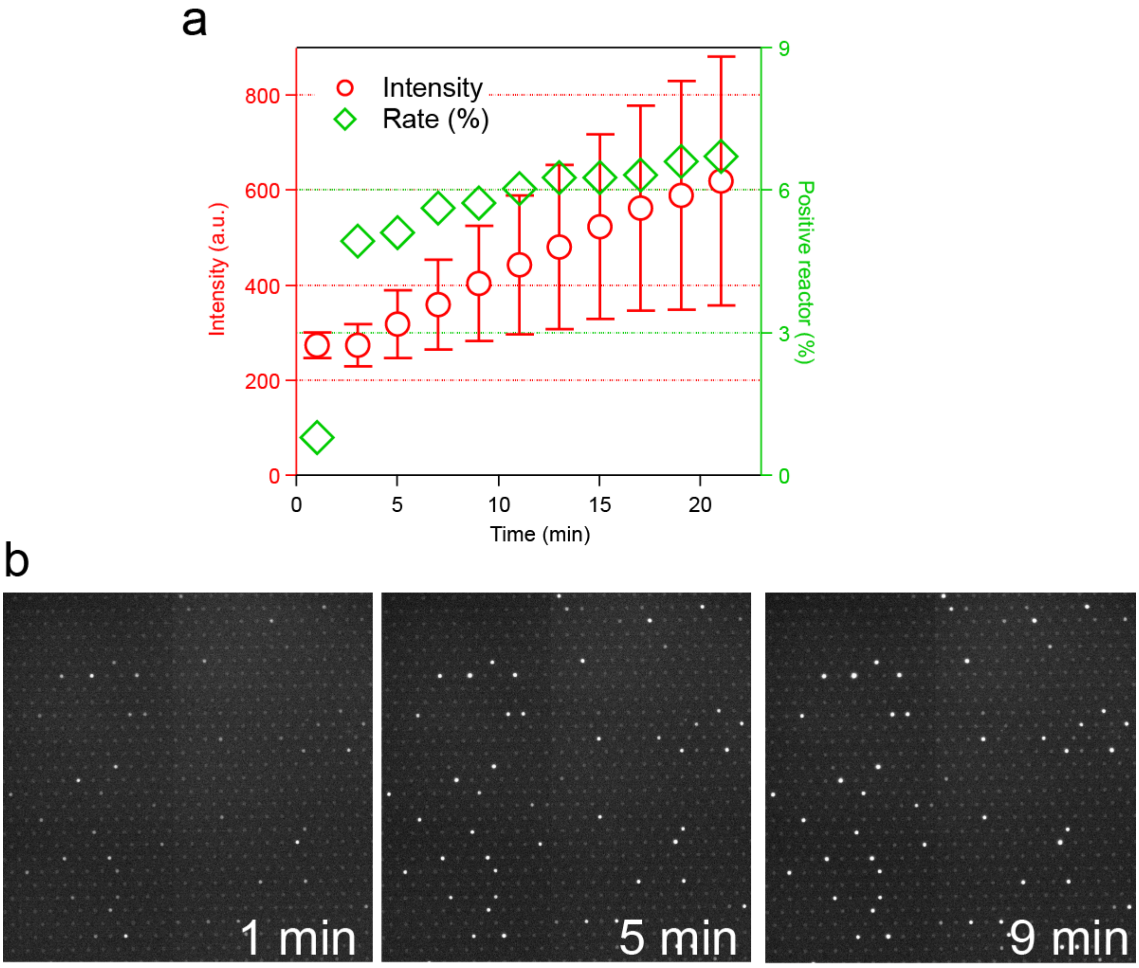

Supplemental Fig. 7. Time course of the fluorescent signal of DIViC

a. The time course of fluorescence signal of positive reactors that displayed a fluorescence signal exceeding the threshold value (mean ± 15 × SD) at the indicated incubation time. Each data point represents an averaged value with standard error. Right axis indicates the corresponding the fraction (%) of the positive reactors. Assays were initiated by mixing samples followed by incubation at room temperature: influenza type A virus (A/PR/8/1934(H1N1)) at 1.0 × 10^6 PFU/mL and MUNANA, and immediately observed using a microscope for time-lapse imaging. **b**. Fluorescent images after 1, 5 and 9 min of incubation.
